# Highly sensitive and rapid identification of coxsackievirus A16 based on reverse transcription multiple cross displacement amplification combined with nanoparticle-based lateral flow biosensor assay

**DOI:** 10.3389/fmicb.2023.1121930

**Published:** 2023-03-08

**Authors:** Jinzhi Cheng, Yu Wang, Yuhong Zhou, Jingrun Lu, Xiaomin Tang

**Affiliations:** ^1^School of Basic Medical Sciences, Guizhou Medical University, Guiyang, China; ^2^Department of Clinical Laboratory, The First People’s Hospital of Guiyang, Guiyang, Guizhou, China; ^3^School of Public Health, The Key Laboratory of Environmental Pollution Monitoring and Disease Control, Ministry of Education, Guizhou Medical University, Guiyang, China; ^4^Laboratory of Bacterial Infectious Disease of Experimental Center, Guizhou Provincial Centre for Disease Control and Prevention, Guiyang, Guizhou, China

**Keywords:** coxsackievirus A16, reverse transcription-multiple cross displacement amplification, hand, foot, and mouth disease, lateral flow biosensor, MCDA-LFB, limit of detection

## Abstract

**Introduction:**

One of the main pathogens responsible for human hand, foot, and mouth disease (HFMD), coxsackievirus A16, has put young children’s health at danger, especially in countries in the Asia-Pacific region. Early quick identification is essential for the avoidance and control of the disorder since there are no vaccinations or antiviral medications available to prevent and manage CVA16 infection.

**Methods:**

Here, we describe the creation of an easy, speedy, and accurate CVA16 infection detection approach using lateral flow biosensors (LFB) and reverse transcriptionmultiple cross displacement amplification (RT-MCDA). A group of 10 primers was developed for the RT-MCDA system in order to amplify the genes in an isothermal amplification device while targeting the highly conserved region of the CVA16 VP1 gene. Then, without requiring any extra tools, RT-MCDA amplification reaction products might well be detected by visual detection reagent (VDR) and LFB.

**Results:**

The outcomes showed that 64°C within 40 min was the ideal reaction setting for the CVA16-MCDA test. Target sequences with <40 copies might be found using the CVA16-MCDA. There was no cross-reaction among CVA16 strains and other strains. The findings demonstrated that the CVA16-MCDA test could promptly and successfully identify all of the CVA16-positive (46/220) samples identified by the traditional real-time quantitative polymerase chain reaction (qRT-PCR) assays for 220 clinical anal swab samples. The whole process, such as the processing of the sample (15 min), the MCDA reaction (40 min), and the documenting of the results (2 min), could be finished in 1 h.

**Conclusion:**

The CVA16-MCDA-LFB assay, which targeted the VP1 gene, was an efficient, simple, and highly specific examination that might be used extensively in rural regions’ basic healthcare institutions and point-of-care settings.

## Introduction

Coxsackievirus A16 (CVA16) is a non-enveloped positive single-stranded RNA molecule of about 7,400 nucleotides (nt), which consists of a lengthy open reading frame (ORF) flanked by 5′ and 3′ untranslated regions (UTRs). It belongs to the genus Enterovirus and the family Picornaviridae (Sickles et al., 1955). In South Africa in 1955, CVA16 was first isolated. For the last several years, enterovirus 71 (EVA71), CVA6, CVA16, CVA10, and other enteroviruses have all been main pathogens responsible for HFMD in young children and infants (Xu et al., 2012; Zhang et al., 2019). Although CVA16 infections often produce minimal symptoms, in previous decades, outbreaks have been related to more serious diseases, including encephalitis, aseptic meningitis, and even fatal cases of CVA16 (Sun et al., 2014; Li et al., 2022). Additionally, CVA16 infection may result in a wide range of medical symptoms, including herpangina and upper and lower respiratory disorders, as well as serious consequences, including meningitis, myelitis, paralysis, encephalitis, and even death (Chen et al., 2019).

According to earlier research, CVA16 infection accounts for around 21% of serious HFMD patients with neurological consequences (Xu et al., 2012). Affected regions face a major public health risk from CVA16 viral infection. No efficient antiviral medications versus CVA16 have been produced to yet. In order to avoid severe cases of HFMD, lower death rates, and stop the spread of the illness in China, it is crucial to create a simple, quick, and reliable identification test for CVA16.

The two main principles of standard CVA16 detection techniques are viral isolation culture and serological testing (Xu et al., 2011). Furthermore, due to complicated processes, poor specificities, and sensitivities, which are either time-consuming or have a high false positive rate, viral isolation and serodiagnosis are not quick and accurate enough. The quantitative real-time fluorescent reverse transcription polymerase chain reaction (qRT-PCR), which is based on nucleic acid amplification, has a significant function in the identification of CVA16 and has fixed the issues with the other techniques stated above (Cui et al., 2013). A qRT-PCR technique, however, needs costly laboratory apparatus and knowledgeable operators, which are not obtainable in resource-limited settings. To resolve these issues, it’s crucial to develop a quick, cheap, and visible CVA16 detection technique.

Multiple cross displacement amplification (MCDA), a novel, inexpensive, quick, easy, and effective isothermal amplification approach identical to LAMP that has been widely employed in the identification of various pathogens, such as bacteria, viruses, and fungi, has been developed to address the limitations of traditional identification (Li et al., 2019; Wang et al., 2021; Chen et al., 2021b). To accomplish targeted amplification using the MCDA approach, 10 primers that bind to 10 different regions of the target sequence must be designed. A number of methods, like turbidimetry, agarose gel, and colorimetric markers (malachite green reagent), have been used to evaluate MCDA results in the past (Wang et al., 2015). However, the findings of these approaches may be easily misunderstood since it is difficult to discriminate between non-specific and specific amplification. The validation of nucleic acid labeled amplification products may be performed using a simple, sensitive, and highly focused lateral flow biosensor (LFB), which was developed and implemented to address these shortcomings (Jiang et al., 2022). Compared to these techniques, LFB is more precise, objective, inexpensive, and simple.

The current study’s objective was to establish a CVA16-MCDA-LFB approach that is quick, portable, sensitive, user-friendly, and can ensure accurate CVA16 identification. Additionally, we used the CVA16-MCDA-LFB assay to identify the rectal swabs in this case, confirming its appropriateness for point-of-care quick detection of HFMD.

## Materials and methods

### Reagents and instruments

Kits for extracting virus RNA nucleic acids were acquired from Tianlong Technology Co., Ltd. (Xi an tian long, ShanXi, China). From Beijing Baitaike Biotech Co., Ltd., a genomic DNA kit for nucleic acid extraction and purification was obtained (Beijing, China). HUIDEXIN Biotech Co., Ltd. (Tianjin, China) supplied the visual detection reagent (VDR), the polymer nanoparticle-based lateral flow biosensor (LFB), and isothermal amplification kits (RNA/DNA Universal). CVA16 qPCR diagnosis kits were acquired from Jin Hao Gene Co., Ltd. (Beijing, China). Nucleic acid concentration and purity were assessed using the Nano-Drop ND-2000 (Beijing, China) at A260/280.

### Viral strains and clinical specimens

During the monitoring of HFMD in Guizhou Province from 2019 to 2021, enterovirus strains were obtained from individuals with HFMD there. To extract enteroviruses, human rhabdomyosarcoma (RD) cells were employed. Specimens that caused a cytopathic effect (CPE) following three passages were deemed positive and kept at −80°C until RNA purification. At 36°C, several bacterial strains were inoculated into the nutrient agar plate. All colonies were obtained independently after 2 days of pure culture. Utilizing a panel of common enteroviruses obtained from HFMD patients and validated by qRT-PCR, this assay’s specificity was assessed. In Table 1, every pathogen was illustrated. Additionally, 220 clinical specimens were taken from patients who had been hospitalized at the First People’s Hospital of Guiyang with suspected HFMD. All clinical samples were examined concurrently by commercial qRT-PCR and MCDA tests in order to assess CVA16-MCDA clinical performance. *The First People’s Hospital of Guiyang’s* Ethics Committee provided its authorization for this investigation (Ethical approval No.G2020-S001). According to the Helsinki Declaration, patients who supplied anal swab specimens provided written informed permission.

**Table 1:** Pathogens used in the current study.

Pathogen	Source of pathogens^a^	No. of strain	CVA16*-*MCDA-LFB result^b^
*Coxsackievirus A16* (CVA16)	GZ2020-06-098(GZCDC)	1	P
*Coxsackievirus A16* (CVA16)	GZ2020-06-0100(GZCDC)	1	P
*Coxsackievirus A16* (CVA16)	GZ2020-07-0119(GZCDC)	1	P
*Coxsackievirus A16* (CVA16)	GZ2020-07-0125(GZCDC)	1	P
*Coxsackievirus A16* (CVA16)	GZ2020-09-0120(GZCDC)	1	P
*Coxsackievirus A16* (CVA16)	GZ2020-09-0123(GZCDC)	1	P
*Coxsackievirus A16* (CVA16)	GZ2020-09-0124(GZCDC)	1	P
*Coxsackievirus A16* (CVA16)	GZ2020-08-023(GZCDC)	1	P
*Coxsackievirus A16* (CVA16)	GZ2021-03-049(GZCDC)	1	P
*Coxsackievirus A16* (CVA16)	GZ2021-04-048(GZCDC)	1	P
*Enterovirus A71* (EVA71)	GZ2021-05-0131(GZCDC)	1	N
*Coxsackievirus A2* (CVA2)	GZ2021-10-0262(GZCDC)	1	N
*Coxsackievirus A4* (CVA4)	GZ2021-04-066(GZCDC)	1	N
*Coxsackievirus A6* (CVA6)	GZ2020-08-089(GZCDC)	1	N
*Coxsackievirus A10* (CVA10)	GZ2021-04-0170(GZCDC)	1	N
*Coxsackievirus A24* (CVA24)	Isolated strains (GZCDC)	1	N
*Coxsackievirus B3* (CVB3)	Isolated strains (GZCDC)	1	N
*Echovirus 30* (ECHO30)	Isolated strains (GZCDC)	1	N
*Enterovirus C96* (EVC96)	Isolated strains (GZCDC)	1	N
*Human rhinovirus (clinical samples)*	GFPH	1	N
*Norovirus (clinical samples)*	GFPH	1	N
*Staphylococcus aureus*	Isolated strains (GFPH)	1	N
*Klebsiella pneumoniae*	Isolated strains (GFPH)	1	N

a GZCDC, Guizhou Provincial Center for Disease Control and Prevention; GFPH: The First People’s Hospital of Guiyang.

b P, positive; N, negative. Only Viral RNA templates from CVA16 could be detected by MCDA-LFB assay, indicating the extremely high specificity of the method.

### Nucleic acid extraction

Following the manufacturer’s recommendations, viral RNA was extracted from 200 μL of cell culture supernatant and anal swabs of suspected HFMD cases using an RNA extraction kit (Xi an tian long, ShanXi, China). Viral RNA was then eluted in 50 μL of nuclease-free water for use instantly or storage at −80°C. According to the manufacturer’s directions, DNA was isolated from each strain utilizing Baitaike DNA extraction kit (Beijing, China). Purity and concentration were then determined using a Nanodrop 2000 (Beijing, China) at A260/280. Before usage, the extracted DNA was kept at −20°C.

### CVA16-MCDA primer design and standard plasmid construction

**Figure 1:**
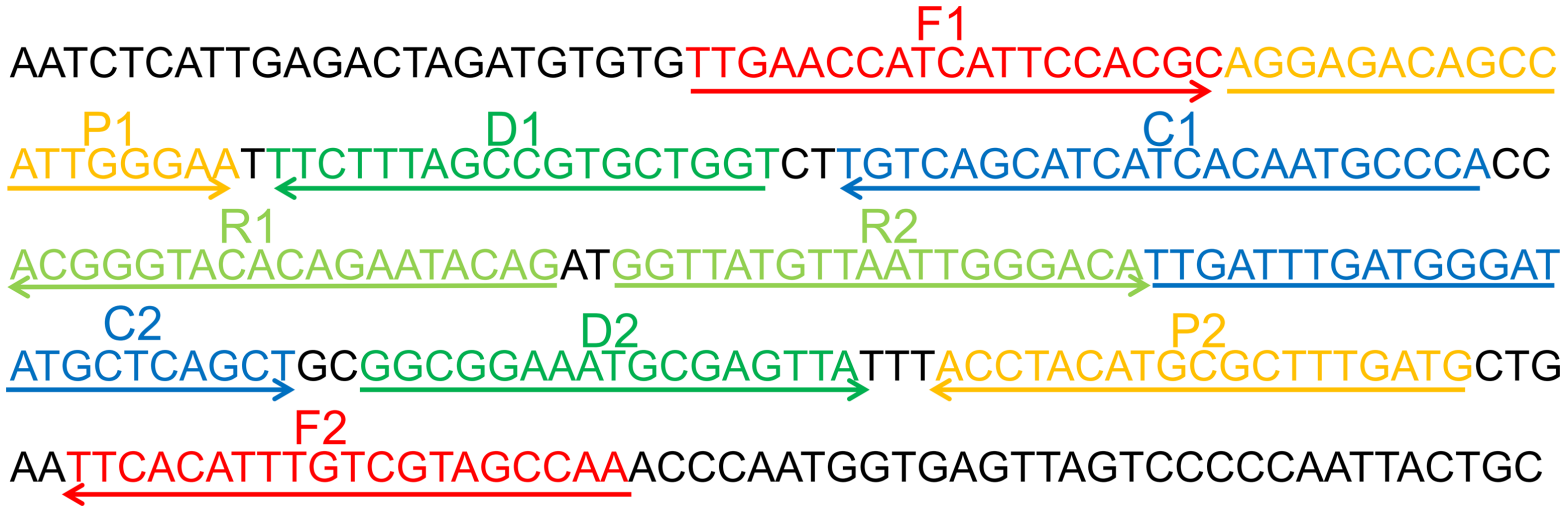
Appropriate primer design for CVA16-MCDA-LFB assay. The nucleotide sequences of the sense strand of VP1 are listed. Right arrows and left arrows indicate sense and complementary sequences that are used.

Primer Explorer 5^1^ was used to create a set of 10 primers depending on the conserved area of the capsid protein VP1 gene (Genbank Accession: GQ279371.1) of CVA16 and the pathway of MCDA (Wang et al., 2015). These primers’ hybrids and hairpin structures were examined utilizing design tools for integrated DNA technologies. Using the BLAST analysis method, the CVA16-MCDA primers’ specificity was validated. Tsingke Biotechnology Co., Ltd. (Kunming, China) with HPLC purification grade was employed to create all primers used. Tsingke Biotechnology Co., Ltd. (Kunming, China) was employed to chemically synthesize and clone a 344-bp VP1 coding region containing the target sequence into the pUC57 plasmid (herein referred to as pUC57-CVA16-VP1), which contained the amplification target of the aforementioned MCDA primers, in order to enhance the MCDA assay and assess the limit of detection. Figure 1 and Table 2 illustrate the sequence details, locations, and alterations of the primers employed in this investigation.

**Table 2:** The primers used in the current study.

Primer/plasmid name^a^	Sequences and modifications (5′–3′)^b^	Length^c^	Gene
F1	TTGAACCATCATTCCACGC	19 nt	VP1
F2	TTGGCTACGACAAATGTGAA	20 nt	
CP1	TGGGCATTGTGATGATGCTGACAAGGAGACAGCCATTGGGAA	42 mer	
CP2	TTGATTTGATGGGATATGCTCAGCTCATCAAAGCGCATGTAGGT	44 mer	
C1	TGGGCATTGTGATGATGCTGACA	23 nt	
C1*	Biotin-TGGGCATTGTGATGATGCTGACA	23 nt	
C2	TTGATTTGATGGGATATGCTCAGCT	25 nt	
D1	ACCAGCACGGCTAAAGAA	18 nt	
D1*	FAM-ACCAGCACGGCTAAAGAA	18 nt	
D2	GGCGGAAATGCGAGTTA	17 nt	
R1	CTGTATTCTGTGTACCCGT	19 nt	
R2	GGTTATGTTAATTGGGACA	19 nt	
The cloned part of VP1 coding sequence (CVA16-VP1 plasmid)	CAGATTAGGCACTGGTGTTGTACCAGCACTGCAAGCCGCGGAGACAGGGGCGTCGTCTAATGCTAGTGACAAGAATCTCATTGAGACTAGATGTGTGTTGAACCATCATTCCACGCAGGAGACAGCCATTGGGAATTTCTTTAGCCGTGCTGGTCTTGTCAGCATCATCACAATGCCCACCACGGGTACACAGAATACAGATGGTTATGTTAATTGGGACATTGATTTGATGGGATATGCTCAGCTGCGGCGGAAATGCGAGTTATTTACCTACATGCGCTTTGATGCTGAATTCACATTTGTCGTAGCCAAACCCAATGGTGAGTTAGTCCCCCAATTACTGC		

a C1*, 5′-labeled with biotin when used in the MCDA-LFB assay; D1*, 5′-labeled with FAM when used in the MCDA-LFB assay.

b FAM, 6-carboxy-fluorescein.

c mer:monomeric unit; nt: nucleotide.

### The standard MCDA-LFB assay

The CVA16-MCDA amplification was conducted in a one step reaction of 25 μL, including the final concentration of each replacement primers (F1 and F2) contains 0.4 μM and 0.8 μM each of amplification primers (C1∗, C2, R1, R2, D1∗, and D2) and 1.6 μM each of cross primers (CP1 and CP2), 12.5 μL of 2 × reaction mix, 1 μL (10 U) Bst 4.0 DNA polymerase, 1 μL (10 U) of AMV reverse transcriptase (only utilized for RNA temperate) and template (1 μL for each standard plasmid, 5 μL RNA for clinical samples), the total volume was made up to 25 μL with double distilled water (ddH_2_O). Also, 1 μL ddH_2_O was functioned as blank control, and virus RNA of EVA71 and CVA6 was employed as a negative control. To run program, the mixtures were incubated for 1 h at 64°C and then for 5 min at 80°C to stop the reaction.

### CVA16-MCDA products detection

The CVA16-MCDA products were identified and validated utilizing three different MCDA detection techniques, including real-time turbidity LA-500 (Eiken Chemical Co., Ltd., Japan), LFB, and colorimetric indicator VDR. Turbidity >0.1 was regarded as a positive outcome for the real-time turbidity approach. The positive reaction solution changed noticeably from colorless to light green when VDR was used, whereas the negative and blank controls maintained colorless. While the CL and TL showed simultaneously during the LFB test, demonstrating positive findings, only the CL was seen during the negative amplification.

### Optimal temperature of CVA16-MCDA assay

The amplification effectiveness of the MCDA reaction system was significantly influenced by temperature. The CVA16-MCDA assay’s optimal amplification temperature range was 61–68°C (at 1°C intervals). As negative controls (NC), amplification mixes containing 5 μL of EVA71 template were employed. As a blank control, 1 μL of double-distilled water (DW) was utilized. The Real-time Turbidimeter LA-500 was used to detect the CVA16-MCDA amplified products, and a threshold value of >0.1 within 1 h was considered a positive response.

### Limit of detection and optimal isothermal amplification time of CVA16-MCDA assay

The initial pUC57-CVA16-VP1 concentration was 4 × 10^8^ copies/μL in order to assess LoD of MCDA assay for CVA16. Then, 10-fold serial dilutions with TE buffer (4 × 10^6^ to 4 × 10^0^ copies) of pUC57-CVA16-VP1 DNA were employed to evaluate LoD of the CVA16-MCDA assays. In the amplification reaction system, 1 μL of the diluted plasmid was supplied as a template. Double distilled water was utilized as the negative control since the plasmid was employed as a template, and the technique for sensitivity testing did not include AMV reverse transcriptase. CVA16-MCDA reaction time was also optimized, and four different times (20–50 min, with 10 min intervals) were compared under identical reaction situations. All amplified products at each time point, including 20, 30, 40, and 50 min, were observed utilizing LFB, and each amplification time was evaluated three times minimum in the present investigation.

### Specificity of CVA16-MCDA assay

Ten CVA16 strains and 13 non-CVA16 pathogens that were amplified under ideal circumstances were used to compare the analytical specificity of the CVA16-MCDA test. LFB was used to observe all MCDA products, and each test was performed at three times minimum.

### Application of CVA16-MCDA-LFB detection in clinical samples

Typically 220 anal swab specimens from *the First People’s Hospital* in Guiyang were obtained to investigate the use of CVA16-MCDA detection in clinical samples. The samples were identified employing CVA16-MCDA-LFB and the qRT-PCR assay, and the outcomes were compared. From Jin hao Gene (Beijing, China), commercial qRT-PCR test kits for enteroviruses were acquired. The 7500 real-time PCR technology (Applied Biosystems, United States) was used to perform the qRT-PCR reactions. All processes were conducted following the manufacturer’s directions. The CVA16-MCDA-LFB determination was conducted, as previously mentioned.

## Results

### Confirmation of effectiveness of MCDA-LFB assay for the detection of CVA16

**Figure 2:**
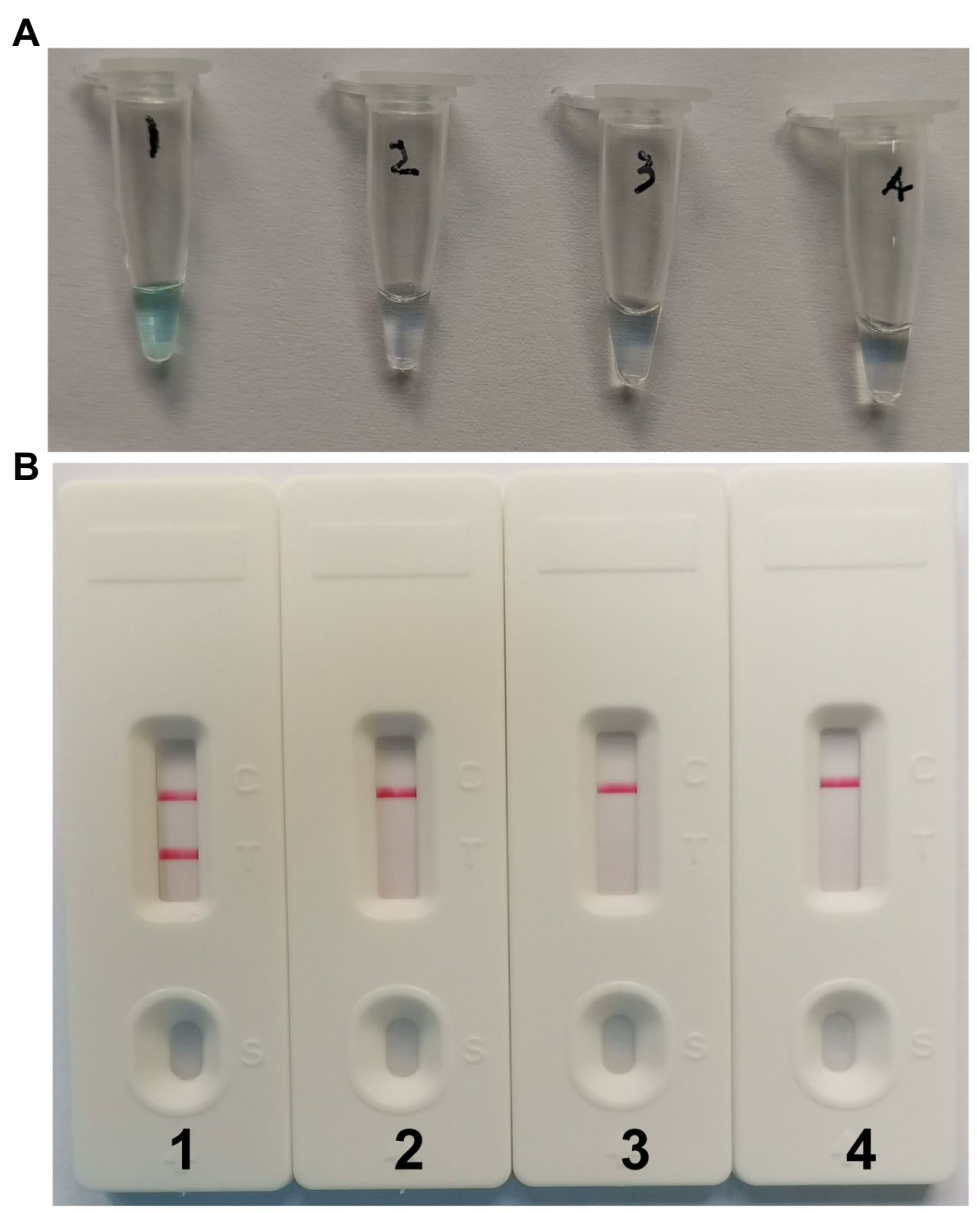
Identification and confirmation of CVA16-MCDA-LFB products. **(A)** The visible color changes of amplification products of CVA16-MCDA-LFB assay were analyzed by VDR. **(B)** The products of CVA16-MCDA-LFB were visually detected with Lateral flow biosensor. Tube 1/biosensor 1, positive amplification of pUC57-CVA16-VP1 DNA; tube 2/biosensor 2, negative control of virus RNA of EVA71; tube 3/biosensor 3, negative control of virus RNA of CVA6; tube 4/biosensor 4, blank control (double-distilled water, DW).

To determine if the MCDA primers (Table 2) for the CVA16 assay are valid. pUC57-CVA16-VP1 DNA was employed as a template for CVA16-MCDA mixes, which were processed for 60 min at a constant temperature of 64°C. Then, two alternative techniques, including VDR and LFB, were utilized to monitor the amplification products. The outcomes revealed that the nucleic acid from CVA16 provided positive results but not those from CVA6, EVA71, and the blank controls (Figures 2A,B). As a result, the CVA16-MCDA primers used in the present investigation to identify the VP1 gene were valid to create the CVA16-MCDA-LFB assay.

### The optimal temperature of CVA16-MCDA-LFB assay

**Figure 3:**
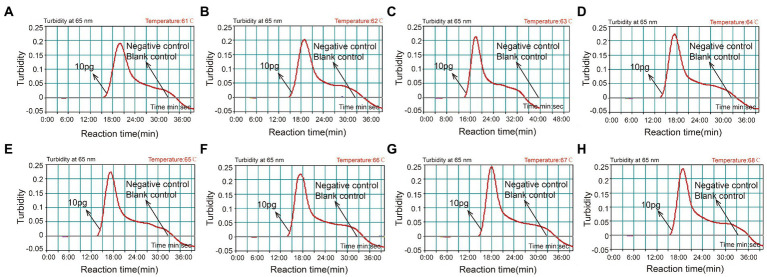
Optimal amplification temperature for CVA16-MCDA-LFB assay. By using a real-time measurement to monitor the turbidity of CVA16-MCDA-LFB reactions. The corresponding curves were displayed in the panels. The negative control was virus RNA of EVA71, and the blank control was sterile double-distilled water. Abscissa represents reaction time (min), ordinate represents turbidity. The threshold value was 0.1, and the turbidity >0.1 was considered as positive amplification. Eight kinetic curves **(A-H)** were generated from 61 to 68°C (1°C intervals), with 4 × 10^6^ copies/μL of pUC57-CVA16-VP1 DNA per reaction.

With 4 × 10^6^ copies/μL of pUC57-CVA16-VP1 DNA as the template, the CVA16-MCDA reaction was conducted from 61 to 68°C to validate the ideal temperature for isothermal amplification. Real-time turbidity LA-500 was employed to observe every reaction. Typically 64°C was determined to be the ideal reaction temperature for CVA16-MCDA amplification since this temperature allowed for the quickest achievement of the 0.1 absorbance threshold value that indicated positive amplification from the CVA16-MCDA reaction (Figure 3). In this investigation, the following CVA16-MCDA-LFB reaction was performed at 64°C.

### Limit of detection and optimized time of MCDA for CVA16 detection

**Figure 4:**
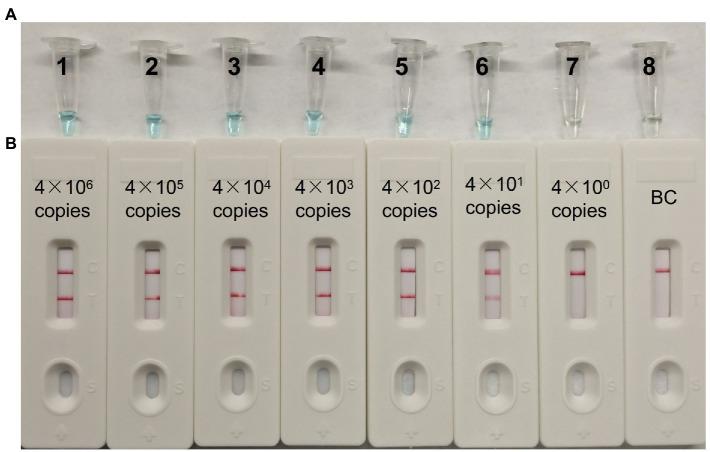
The detection limit of CVA16-MCDA-LFB assays. Two measurement techniques, including **(A)** a colorimetric indicator (VDR) and **(B)** lateral flow biosensor. A series of dilutions (4 × 10^6^–4 × 10^0^ copies) of pUC57-CVA16-VP1 DNA and a blank control (BC) double-distilled water were operated according to standard MCDA-LFB reactions.

A serial dilution of the pUC57-CVA16-VP1 (4 × 10^6^, 4 × 10^5^, 4 × 10^4^, 4 × 10^3^, 4 × 10^2^, 4 × 10^1^ and 4 × 10^0^ copies per microliter) was employed in MCDA assays to define CVA16-MCDA-LFB assay LoD. When the dilution passed 4 × 10^1^ copies/μL, as illustrated in Figure 4, MCDA tubes displayed colorless and only control band in LFB. Bright blue MCDA tubes were shown for other dilutions, and two red bands—one representing the control line and the other representing the test line—were noticed in the LFB.

**Figure 5:**
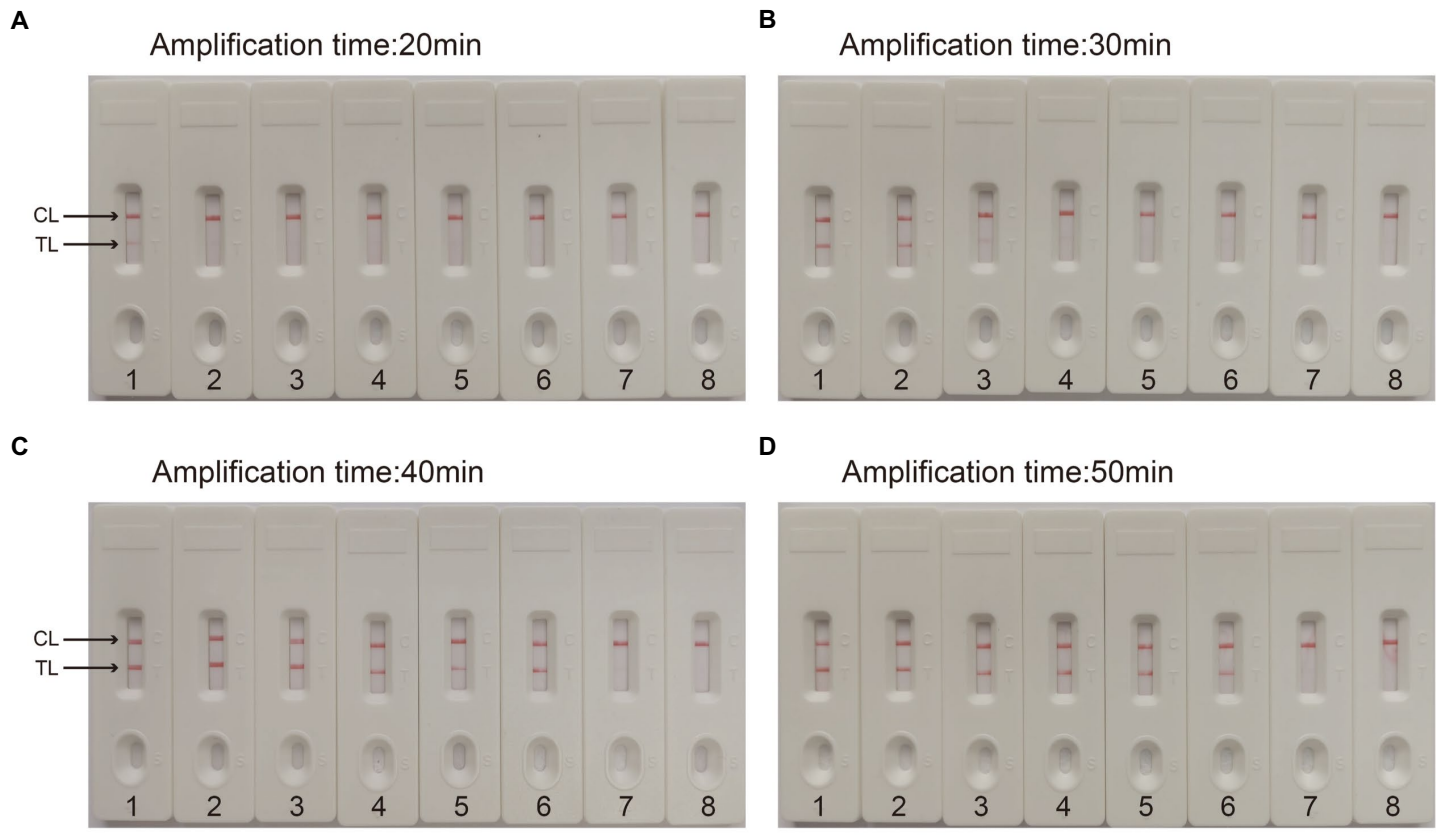
Optimal detection time required for CVA16-MCDA-LFB assay. Four different reaction times [**(A)** 20 min; **(B)** 30 min; **(C)** 40 min; **(D)** 50 min] were evaluated at 64°C. Biosensors 1–7 represent pUC57-CVA16-VP1 DNA levels of 4 × 10^6^ to 4 × 10^0^ copies per reaction, respectively; 8 represents a blank control double-distilled water.

**Figure 6:**
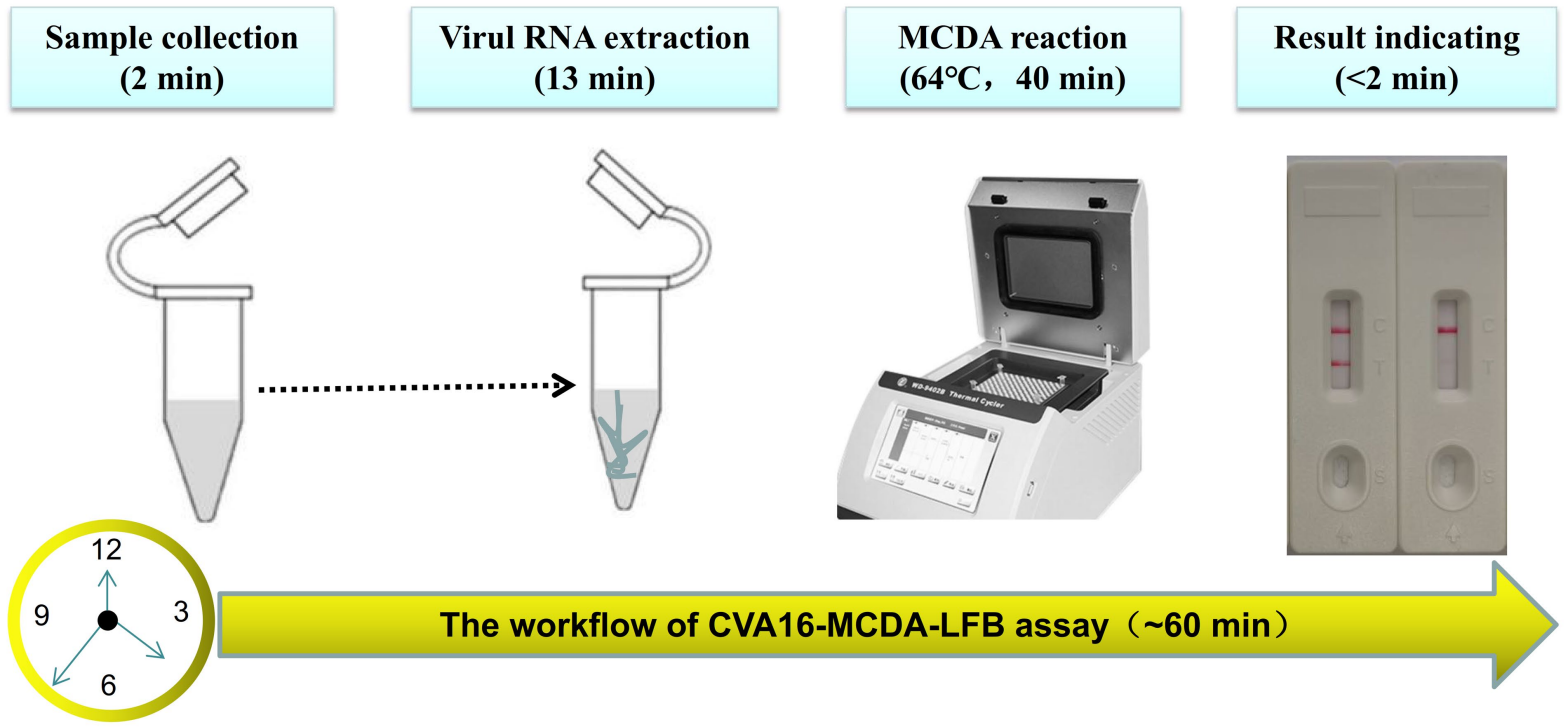
The workflow of CVA16-MCDA-LFB assay. Four steps, including sample collection (2  min), Virul RNA extraction (13  min), MCDA reaction (40  min) and result reporting (<2  min), are required for the CVA16-MCDA-LFB assay, and the total procedure can be completed within 60  min.

For the CVA16-MCDA-LFB assay, four amplification times (20, 30, 40, and 50 min) were examined, respectively, at 64°C to determine the best amplification time. With LFB, the amplification products were observed. The outcomes showed that the pUC57-CVA16-VP1 LoD level (4 × 10^1^ copies/μL) was evaluated while the amplification lasted 40 and 50 min (Figure 5). When the isothermal reaction was performed for 40 min at 64°C, the lowest template level (4 × 10^1^ copies) showed two red bands (Test line and Control line). So, the ideal conditions for the remaining CVA16-MCDA-LFB tests in the present investigation were a reaction temperature of 64°C and an amplification period of 40 min. Thus, the whole diagnostic procedure of the CVA16-MCDA-LFB assay, including Sample collection (2 min), Virul RNA extraction (13 min), MCDA reaction (40 min) and result reporting (<2 min), can be completed within 60 min (Figure 6).

### Specificity of MCDA-LFB for CVA16 detection

**Figure 7:**
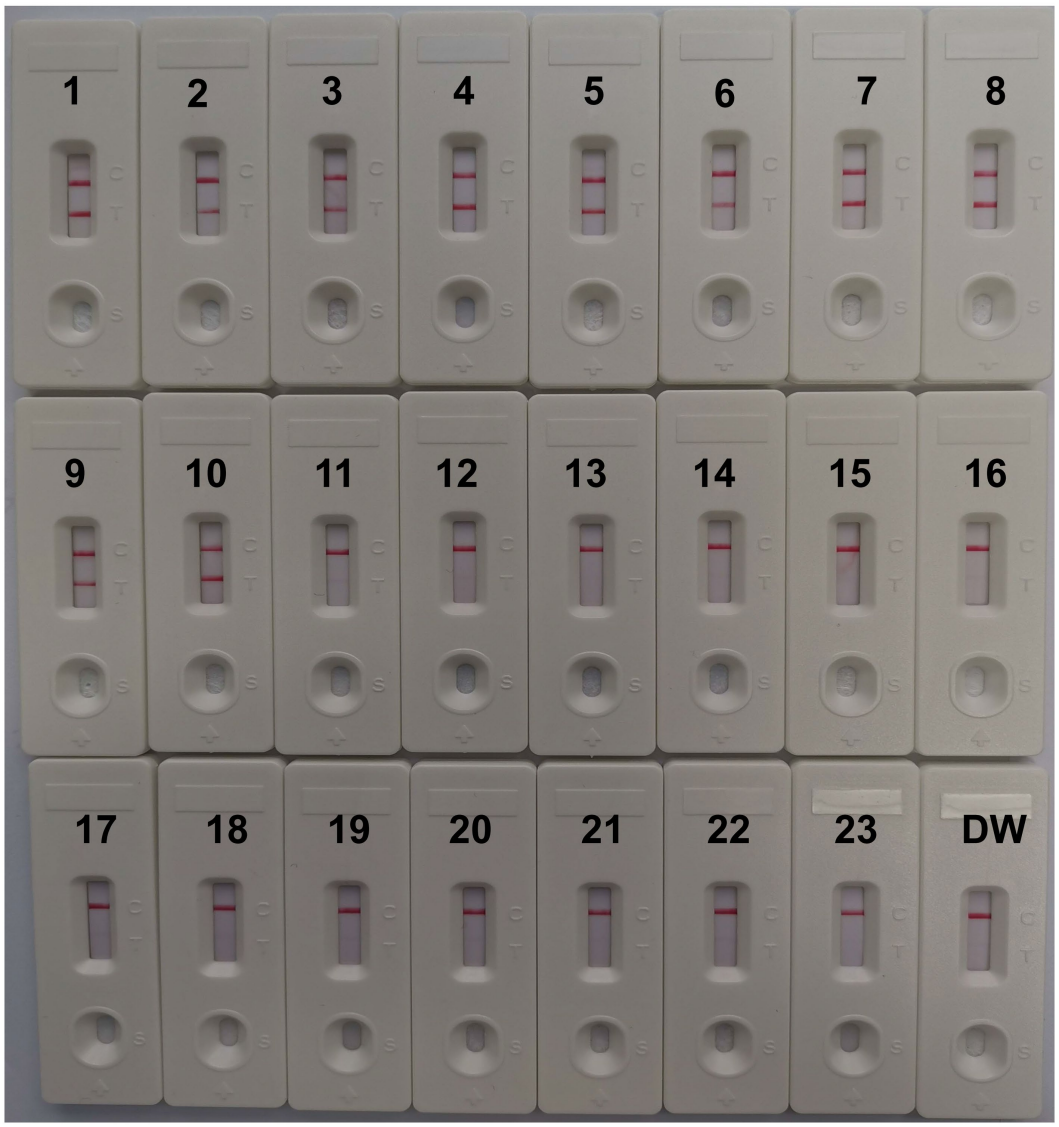
Specificity of LFB assays detecting CVA16-MCDA-LFB products. The CVA16-MCDA-LFB assay was evaluated with different genomic DNA as templates. Both the control line and the test line were visible in LFB for all CVA16, and only the control line was appeared in non-CVA16. 1–10, CVA16 isolated strains; 11, EVA71 isolated strains; 12, CVA2 isolated strains; 13, CVA4 isolated strains; 14, CVA6 isolated strains; 15, CVA10 isolated strains; 16, CVA24 isolated strains; 17, CVB3 isolated strains; 18, ECHO30 isolated strains; 19, EVC96 isolated strains; 20, Human rhinovirus clinical samples; 21, Norovirus clinical samples; 22, Staphylococcus aureus isolated strains; 23, Klebsiella pneumoniae isolated strains; 24, a blank control double-distilled water (DW).

CVA16-MCDA-LFB test specificity was determined using 13 non-CVA16 pathogens and 10 isolated CVA16 strains (Table 1). The MCDA-LFB detection was conducted in the ideal situations indicated above. Employing LFB, two red lines were visible at the locations of Test line and Control line for the CVA16 strain, but only one line was observed at the position of C line for all non-CVA16 pathogens and the blank control (Figure 7), indicating that the CVA16-LAMP-LFB assay had 100% specificity.

### Validation with clinical samples

Two hundred twenty suspected HFMD infection anal swab specimens were concurrently identified using CVA16-MCDA-LFB and real-time PCR (LoD: 500 copies) to further validate the use of CVA16-MCDA-LFB as a useful approach for CVA16 identification in clinics. Typically 46 of the 220 anal swab samples were positive for CVA16. Outcomes from the CVA16-MCDA-LFB assay perfectly matched those from the real-time PCR assay (Table 3). These results showed that the CVA16-MCDA-LFB assay provides a quick, accurate, and sensitive detection of CVA16 and can be used as a potential screening tool for CVA16 in a clinical and basic laboratory. It is particularly suitable for screening HFMD patients at the early stages of infection.

**Table 3:** Comparison of real-time PCR and CVA16-MCDA-LFB methods to identify CVA16 in clinical samples.

Detection methods	Nasopharyngeal swab samples (*n* = 220)		Amplification time (min)	Sensitivity (%)	Specificity (%)
	Positive	Negative			
real-time PCR	46	176	100	100	100
CVA16-MCDA-LFB assay	46	176	40	100	100

## Discussion

One of the most prevalent diseases in the world is HFMD. According to studies by Zhuang et al. (2015) and Esposito and Principi (2018), it disproportionately affects young children under the age of five. The Chinese Ministry of Health closely monitors it and has classified it as a category III notifiable infectious illness since 2008. (Nishimura and Shimizu, 2012). The two main pathogens of HFMD are enterovirus 71 (EVA71) and CVA16. Since the EVA71 vaccination has been available since late 2016, the proportion of HFMD cases related to EVA71 has significantly dropped (Takahashi et al., 2016; Han et al., 2020). Nonetheless, the CVA16 epidemic persists, and more than 22% of all HFMD cases are recorded annually in China (Yi et al., 2021). As a result, CVA16 is a significant public health issue, and there is no available medication or vaccination to prevent it. Therefore, it’s crucial to differentiate CVA16 quickly, precisely, and sensitively in order to stop and manage HFMD epidemics. Traditional detection techniques, such as viral isolation culture and serological testing, along with PCR-based technologies, often cannot meet the necessities for rapid identification in terms of time and sensitivity. Therefore, it is necessary to create a new technique to easily, quickly, sensitively, and precisely detect and diagnose CVA16.

Here, we describe the use of LFB and MCDA to identify CVA16. The MCDA is an isothermal amplification technique that does not need specialized tools or expert knowledge, only a basic water bath or heater (Jiao et al., 2019). A total of 10 specific primers were created using the MCDA-LFB approach to identify 10 distinct regions of the target sequence. We have chosen the highly conserved area of VP1 gene, which is very crucial for the immunogenicity of CVA16 vaccine since the primary neutralizing epitopes are situated in this region (Shi et al., 2013). Amplification products could be detected by the LFB labeled with FAM and biotin; it took about 2 min to read the LFB’s findings. Compared to MCDA methods used in earlier studies, the CVA16-MCDA method identified outcomes in LFB, which excludes the demands of specific reagents (e.g., pH indicators), difficult processes (e.g., electrophoresis), and expensive devices (e.g., real-time turbidity), was quick, sensitive, simple for using, and not prone to error (Wang et al., 2018; Zhao et al., 2019; Chen et al., 2021a).

In the CVA16-MCDA-LFB test, we altered the reaction temperature and reaction time to find the perfect situations. At 64°C, CVA16-MCDA-LFB has a greater amplification efficiency than it has at other temperatures. Additionally, because of CVA16-MCDA-great LFB’s sensitivity, the findings demonstrated that it was possible to detect the LoD level of 4 × 10^1^ copies/μL of pUC57-CVA16-VP1 during continuous 40-min amplification. This suggests that the MCDA-LFB technique is a quick, accurate, and simple way to find small quantities of CVA16. The CVA16-MCDA-LFB test has a high specificity in addition to its excellent sensitivity. The positive findings for the CVA16-MCDA-LFB assay specificity test were obviously obtained from CVA16 strains but not from non-CVA16 strains. More crucially, we were able to identify Nasopharyngeal swab specimens from clinics using the CVA16-MCDA-LFB test. Compared with real-time PCR method, CVA16-MCDA-LFB technique is more time-saving, with 100% positive and 100% negative rates, real-time PCR needs 3–4 h during the whole process (Guney et al., 2003; Cui et al., 2013). However, even with sample preparation, the CVA16-MCDA-LFB test procedure’s overall detection time was <1 h. It was shown that our established approach for detecting CVA16 is quicker than methods based on culture and real-time PCR. Antibiotic exposure may be decreased when doctors are able to provide targeted medications to patients more rapidly since the CVA16-MCDA-LFB test can reveal findings in only 1 h. This makes this technique potentially valuable for detecting target pathogens.

The CVA16-MCDA-LFB technique in the present investigation also has significant restrictions. First, the recently discovered CVA16-MCDA-LFB detection is a qualitative evaluation of the pathogen and is unable to measure the pathogen quantity in specimens. The MCDA-LFB assay will be used in a more accurate investigation to assess CVA16 in clinical samples. Second, for LFB detection, CVA16-MCDA amplifications must be eliminated from the reaction tube since carry-over contamination is a major issue with this approach. However, spraying 70% ethanol and sodium hypochlorite solution is also useful in avoiding DNA contamination after identification has been finished. Additionally, MCDA is an isothermal amplification method that needs many primer pairs; contamination may easily happen and provide false findings. We will thus address the aforementioned issues in subsequent investigations.

## Conclusion

A visual, quick, easy, and sensitive CVA16-MCDA-LFB assay based on the VP1 gene was successfully created in the current investigation to detect CVA16. When compared to real-time PCR molecular diagnostic tests, the MCDA-LFB assay avoids complex procedures and does not demand costly equipment or experienced technical staff. The LoD of the new test for detecting CVA16 in an isolated sample was as low as 40 copies, indicating that the MCDA-LFB assay was very sensitive. More significantly, the new test may decrease detection time and support clinicians in providing targeted treatments for patients with HFMD more rapidly, particularly in resource-poor regions.

## Data availability statement

The original contributions presented in the study are included in the article/supplementary material, further inquiries can be directed to the corresponding authors.

## Ethics statement

The study was approved by the Human Ethics Committee of *the First People’s Hospital of Guiyang* (approval no. G2020-S001) and acts following the Declaration of Helsinki. The monitoring stations deleted all identifying information from the people suspected of having HFMD infection prior to obtaining the samples/isolates and conducting the study. The First People’s Hospital in Guiyang’s Human Ethics Committee waived the patient’s informed consent.

## Author contributions

JC, YW, XT, and JL conceived and designed the experiments. JC, YZ, YW, and JL conducted the trials. JC, YW, and XT analyzed the data. YW, JC, YZ, and XT wrote the manuscript. All authors contributed to the article and approved the submitted version.

## Funding

This work was funded by a grant from the National Natural Science Foundation of China (grant no. 81860594), Zhu Ke He Tong [2021]-43-25 and Zhu Ke He Tong [2020]-10-6 from Science and Technology Department of Guiyang city of Guizhou Province, Qian Ke He [2018]1094, Qian Ke He Zhi Cheng [2021] yi ban 440 from Science and Technology Department of Guizhou Province, and The Project for the growth of Young Scientific and technological talents in ordinary Colleges and Universities in Guizhou Province (Qian jia he KY zi [2022] No. 231).

## Conflict of interest

The authors declare that the research was conducted in the absence of any commercial or financial relationships that could be construed as a potential conflict of interest.

## Publisher’s note

All claims expressed in this article are solely those of the authors and do not necessarily represent those of their affiliated organizations, or those of the publisher, the editors and the reviewers. Any product that may be evaluated in this article, or claim that may be made by its manufacturer, is not guaranteed or endorsed by the publisher.

## Footnotes

^1^https://primerexplorer.jp
